# Oxytetracycline and Streptomycin Resistance Genes in *Xanthomonas arboricola* pv. *pruni*, the Causal Agent of Bacterial Spot in Peach

**DOI:** 10.3389/fmicb.2022.821808

**Published:** 2022-02-25

**Authors:** Austin Herbert, C. Nathan Hancock, Brodie Cox, Guido Schnabel, Daniela Moreno, Renato Carvalho, Jeffrey Jones, Matthew Paret, Xueqing Geng, Hehe Wang

**Affiliations:** ^1^Edisto Research and Education Center, Clemson University, Blackville, SC, United States; ^2^Department of Biology and Geology, University of South Carolina Aiken, Aiken, SC, United States; ^3^Department of Plant and Environmental Sciences, Clemson University, Clemson, SC, United States; ^4^Department of Plant Pathology, University of Florida, Gainesville, FL, United States; ^5^North Florida Research and Education Center, University of Florida, Quincy, FL, United States; ^6^School of Agriculture and Biology, Shanghai Jiao Tong University, Shanghai, China

**Keywords:** antibiotic resistance, plant disease, bacterial pathogen, stone fruits, horizontal gene transfer, transposon, plasmid

## Abstract

*Xanthomonas arboricola* pv. *pruni* (*Xap*) causes bacterial spot, a major worldwide disease of *Prunus* species. Very few chemical management options are available for this disease and frequent applications of oxytetracycline (OTC) in the United States peach orchards have raised concerns about resistance development. During 2017–2020, 430 *Xap* strains were collected from ten peach orchards in South Carolina. Seven OTC-resistant (OTC*^R^*) *Xap* strains were found in 2017 and 2020 from four orchards about 20–270 km apart. Interestingly, the seven strains were also resistant to streptomycin (STR). Six strains grew on media amended with ≤100 μg/mL OTC, while one strain, R1, grew on ≤250 μg/mL OTC. Genome sequence analysis of four representative OTC*^R^* strains revealed a 14–20 kb plasmid carrying *tetC*, *tetR*, and *strAB* in each strain. These three genes were transferable to *Xanthomonas perforans* via conjugation, and they were PCR confirmed in all seven OTC*^R^ Xap* strains. When *tetC* and *tetR* were cloned and expressed together in a sensitive strain, the transconjugants showed resistance to ≤100 μg/mL OTC. When *tetC* was cloned and expressed alone in a sensitive strain, the transconjugants showed resistance to ≤250 μg/mL OTC. *TetC* and *tetR* expression was inducible by OTC in all six wild-type strains resistant to ≤100 μg/mL OTC. However, in the R1 strain resistant to ≤250 μg/mL OTC, *tetR* was not expressed, possibly due to the presence of Tn*3* in the *tetR* gene, and in this case *tetC* was constitutively expressed. These data suggest that *tetC* confers OTC resistance in *Xap* strains, and *tetR* regulates the level of OTC resistance conferred by *tetC*. To our knowledge, this is the first report of OTC resistance in plant pathogenic xanthomonads.

## Introduction

*Xanthomonas arboricola* pv. *pruni* (*Xap*) causes bacterial spot, a major worldwide disease on stone fruits (e.g., peach, plum, nectarine, apricot, and cherry) and almond (Garita-Cambronero et al., 2018). *Xap* is an economically important pathogen for the peach industry worldwide and especially for producers in the southeastern United States. The disease produces substantial expenses for disease management throughout the growing season. Producers start spraying during “late dormancy” and continue the practice in 7–10-day intervals throughout the season. Despite these efforts, producers experience millions of dollars in yield loss at harvest in form of unmarketable fruit. Fruit infections result in damage that can affect majority of the fruit surface. Under favorable conditions, bacterial spot can affect 100% of the fruit in peach orchards (Stefani, 2010; Pothier et al., 2011a,b). Foliar infections may result in early defoliation, which leads to weakened trees that may impact winter hardiness. Twig infections lead to twig cankers (also called “spring cankers”), often observed at the beginning of the growing season, which are considered the major source of initial inoculum for leaf and fruit infection (Garita-Cambronero et al., 2018; Cox, 2020).

In United States, conventional management strategies for bacterial spot rely heavily on antibacterial sprays, primarily copper-based compounds and oxytetracycline (OTC) (Yang et al., 2013; Lamichhane, 2014). Cultural practices include optimizing fertilizer and water input, eliminating infected tissues, and intercropping; they are most effective when paired with antibacterial sprays (Yang et al., 2013; Lamichhane, 2014; Giovanardi et al., 2017; Garita-Cambronero et al., 2018). A few disease tolerant cultivars (i.e., Clayton and Candor) are available, however, these cultivars still develop bacterial spot symptoms under high disease pressure and they lack favorable fruit quality necessary for maximizing marketability (Yang et al., 2013; Aranzana et al., 2019).

Disease control with conventional bactericides can be a challenge. Even with weekly applications of copper and OTC, bacterial spot incidence can remain high in southeastern peach orchards under conducive environmental conditions (warm and rainy). Recently, *Xap* strains with different levels of copper tolerance were identified and they were found to reduce the efficacy of copper compounds used to control bacterial lesions on peach leaves; the copper rates applied at “shuck split” [599 μg/ml Metallic Copper Equivalent (MCE)] and “summer cover spray” (120 μg/ml MCE) were only able to reduce the bacterial spot incidence caused by the copper-sensitive strain, but not by the copper tolerant strains (Cox, 2020). OTC resistance in *Xap* has not been previously reported but the emergence of resistance is of grave concern since no other chemical control options are available.

Oxytetracycline is a tetracycline that exhibits a broad range of bacteriostatic activity on both Gram-negative and Gram-positive bacteria (Chopra and Roberts, 2001; Christiano et al., 2010). Once inside bacterial cells, OTC binds with high affinity to the 30S subunit and prevents the amino-acyl tRNA from binding to the A site of the ribosome, therefore preventing translation initiation and protein elongation (Chopra and Roberts, 2001). The efficacy of OTC for disease control is directly affected by the frequency and dosage of application, and environmental factors such as precipitation, sunlight (particularly UV light), and temperature (Christiano et al., 2010). Some peach orchards are treated almost weekly with OTC [150 ppm (μg/mL)] during the spring and summer months (Capasso, 2016). However, due to its broad-spectrum activity, the prolonged use of OTC spanning several decades may enrich bacterial communities with antibiotic resistant phenotypes in the phylloplane (Schnabel and Jones, 1999; Vidaver, 2002; Levy and Marshall, 2004).

Tetracyclines have been clinically relevant since the 1940s, and their long-term extensive clinic use for treating bacterial infections has contributed to the emergence and selection of tetracycline resistance genes among human and environmental bacteria (Roberts, 1996; Chopra and Roberts, 2001; Kyselková et al., 2015; Grossman, 2016). There are over 37 genes which are independently or cooperatively able to confer tetracycline resistance, and they are grouped by the biochemical pathways used by their proteins to provide resistance, including tetracycline efflux, ribosomal protection, modification of the tetracycline molecule, and modification of the tetracycline target site (Chopra and Roberts, 2001; Grossman, 2016; Sundin and Wang, 2018; Møller et al., 2020). Tetracycline efflux is the most common resistance mechanism; nearly 30 genes encode for tetracycline-specific efflux pumps, a family of membrane-bound pumps which readily sequester tetracyclines to the periplasm in exchange for a proton (Grossman, 2016; Møller et al., 2020). Several of these efflux genes (*tetA, tetC, tetD, tetE, tetG, tetH, and tetI*) are flanked upstream by a corresponding repressor *tetR*, which is oriented in the reverse direction and divergently transcribed (Chopra and Roberts, 2001). The *tetR* gene encodes a DNA binding protein, a repressor that inhibits transcription of the efflux gene in the absence of tetracycline (Hillen and Berens, 1994; Aleksandrov et al., 2009). Tetracycline efflux genes are commonly acquired through horizontal gene transfer (HGT), as they are often associated with mobile genetic elements with broad host range (Partridge et al., 2018). Furthermore, the tetracycline efflux genes are often co-inherited with other antibiotic resistance genes (e.g., *strAB*, *sulI*, and *sulII*) linked to the same mobile genetic element (Yamaguchi et al., 1990; Schnabel and Jones, 1999; Guardabassi et al., 2000; Shin et al., 2015).

Oxytetracycline was first registered as a pesticide in 1974 (EPA, 1993). Despite the long-term usage in agriculture, the previous reports of OTC resistance in phytopathogens have been limited to *Pseudomonas syringae* pv. *syringae* (resisting up to 500 μg/mL) and *Agrobacterium tumefaciens* (resisting >20 μg/mL), and the genetic determinants of the resistance were only characterized in *A. tumefaciens* as *tetA* and *tetR* (Spotts and Cervantes, 1995; Luo and Farrand, 1999; Hwang et al., 2005). Capasso (2016) surveyed for OTC resistance in *Xap* and epiphytic bacteria in the Pennsylvania peach orchards, and only found OTC resistance in epiphytic bacteria (minimum inhibitory concentration: 450 μg/mL), which included uncharacterized species under several genera (*Pantoea*, *Rahnella*, *Pseudomonas*, or *Xanthomonas*) that screened positive for one of the three tetracycline efflux genes (*tetA, tetB*, and *tetC*). These OTC*^R^* (OTC-resistant) epiphytic bacteria were isolated from peach leaves with or without OTC sprays, which indicates that bacterial epiphytes in the phylloplane of peach orchards could serve as reservoirs for tetracycline resistance genes regardless of selection pressure from OTC sprays.

We hypothesized that the prolonged usage of OTC may have led to the emergence of resistant *Xap* strains in peach orchards of southeastern United States. Therefore, in this study, we surveyed South Carolina peach orchards during 2017–2020 and identified the first OTC*^R^ Xap* strains, which were also resistant to streptomycin (STR*^R^*). The genetic determinants for resistance to these antibiotics were identified and characterized via PCR, whole genome sequencing, and gene functional analysis.

## Materials and Methods

### Isolation of *Xap*

During 2017–2020, symptomatic leaves and fruit were collected from a total of ten peach farms from six counties across South Carolina; symptomatic twig samples were also collected during 2018 and 2019 (Supplementary Table 1). Each sample was surface sterilized and *Xap* was isolated from the margins of bacterial spot lesions onto sucrose peptone agar (SPA, Willbrinks medium) (Schaad et al., 2001) and incubated at 25°C for 3–4 days; colonies with *Xap* morphology were then purified and confirmed with qPCR using *Xap*-specific primers (Palacio-Bielsa et al., 2011). All the strains were stored in cryogenic storage media (Burr, 1993) in −80°C. The suspensions (∼10^8^ CFU/mL in 0.01% of Silwet^®^ L-77) of the OTC*^R^* strains and a subset of the sensitive strains were sprayed onto the leaves of 1-year-old “CaroRed” trees in the greenhouse. Leaves sprayed with water served as control. Bacterial spot symptoms on inoculated peach leaves were evaluated weekly for up to 4 weeks after inoculation.

### Chemical Screening

All *Xap* strains were screened for resistance to OTC. Isolates grown overnight on nutrient agar (NA, Thermo Fisher Scientific, Waltham, MA, United States) were suspended in 1x phosphate-buffered saline (PBS) to an OD_600_ value of 0.1 (∼1 × 10^8^CFU/ml). Then, 10 μL of each suspension was dropped on to NA amended with 25 μg/mL of OTC using oxytetracycline hydrochloride (>99% purity, Sigma Aldrich; St. Louis, MO, United States) and incubated for 4 days at 28°C. The bacterial suspension dropped on NA without OTC was included as control. There were two experiments with two technical replicates for each strain. Compared to the growth on the NA control plates, the percentage of growth of each strain on OTC-amended NA was recorded at 3 and 4 days after incubation. Those strains with growth on plates amended with 25 μg/mL OTC were considered OTC*^R^*. The OTC*^R^* isolates were further screened on NA and NA amended with OTC at 25, 50, 75, 100, and 250 μg/mL and STR at 100, 200, and 300 μg/mL. There were two technical replicates per treatment and the whole experiment was independently replicated three times.

### PCR Screen for Antibiotic Resistance Genes

Oxytetracycline-resistant isolates were subjected to PCR screens for the potential resistance genes. One-to-two-day old culture was suspended in 1x PBS and used as DNA template for qPCR with *tetC* primers that target partial *tetC* sequence (Table 1; Fan et al., 2007) and SsoAdvanced Universal SYBR Green Supermix (Bio-Rad Laboratories, Hercules, CA, United States) at 98°C for 3 min and 35 cycles of 98°C for 15 s, 60°C for 20 s and 72°C for 45 s, and a melt curve analysis from 65 to 95°C with an increment of 0.5°C every 5 s. *tetR* was amplified with the tetR-F and tetR-R primers (Table 1) using a GoTaq G2 Flexi DNA polymerase kit (Promega, Madison, WI, United States) with a temperature cycle of 95°C for 2 min, then 34 cycles of 95°C for 15 s, 57°C for 20 s and 72°C for 30 s, with a final extension at 72°C for 5 min. The *strA* gene was amplified with the strAP-F and strAT-R primers and *strB* with the strBP-F and strBT-R primers (Table 1), using the above mentioned GoTaq kit with a temperature cycle of 95°C for 5 min, then 34 cycles of 95°C for 15 s, 55°C (*strA*) or 56°C (*strB*) for 20 s, 72°C for 90 s, followed by a final extension of 72°C for 5 min.

**TABLE 1:** Bacterial strains, plasmids, and primers used in this study.

Bacteria	Relevant characteristics	Source
*Xanthomonas perforans* GEV1001	Rif^R^, Cu^R^	This study
Stellar competent cells	HST08	Takara Bio
*Xanthomonas arboricola* pv. *pruni* 2WF9	Rif^R^	This study
Δ2WF9-tetCR	pBBR1-MCS-2-tetCR	This study
ΔGEV1001-tetC	pBBR1-MCS-2-tetC	This study
**Plasmids**		
pGEM-T Easy	Amp^R^, lacZ cloning site	Promega
pBBR1-MCS-2	Kan^R^, lacZ cloning site	Addgene
pRK2013	Kan^R^	Addgene
**Primers**		
tetC-F	CTTGAGAGCCTTCAACCCAG	Fan et al., 2007
tetC-R	ATGGTCGTCATCTACCTGCC	Fan et al., 2007
tetR-F	TTCGACGCCAAGGGATGAC	This study
tetR-R	CGTTCAAGACCGCCGATGA	This study
gyrA-F	AGGGTAACTTCGGTTCGGTC	This study
gyrA-R	CGGTTCCTGTTCCTTCTCGT	This study
tetCP-F	TCGCGAATTCTCATGTTT GACAGCT	This study
tetCT-R	TTGGCTCCAATTCTTGGAG TGGTGA	This study
strAP-F	TCATCAGAAAACTGAAGG AACCTC	This study
strAT-R	GAGTCCCGTCTGGCAATGAAA	This study
strBP-F	TTTCCTGCTCATTGGCACGTTT	This study
strBT-R	GAGGGCGAAATCCTACGCTA	This study
tetR-xba1	ATCTCTAGAGGAGGG GTTGCCCTCGATGT	This study
tetR-kpn1	ATCGGTACCTTGGCTCC AATTCTTGGAGTGGTGA	This study
tetR-All-F	CGGTGCCTGACTGCGTTAG	This study
5276R	GAAAATCGTCTACGAAGGCGGTC	This study
3708F	TCGATTCAATGGAGGTTCCTTCA	This study
FP1	CGTCGACGGCCTGGGCGA	Strayer et al., 2016
RP1	CCGGTGCCTGCGCCTGGA	Strayer et al., 2016
Xp-P	/56-FAM/CGGGCAAGG/ZEN/AGC CATCGCCTGT/3IABkFQ/	Strayer et al., 2016
Xap-2F	TGGCTTCCTGACTGTTTGCA	Palacio-Bielsa et al., 2011
Xap-2R	TCGTGGGTTCGCTTGATGA	Palacio-Bielsa et al., 2011
Xap-2P	/56-FAM/TCAATATCT/ZEN/GTGCGT TGCTGTTCTCACGA/3IABkFQ/	Palacio-Bielsa et al., 2011
pXap-41-F	ATGAAAAAGCTCTCTATCGCCCT	This study
pXap-41-R	TTCCCCCTTCTTGTTGAAATCGA	This study
pMDR_4KB_F	CCTTCATTCCGACACGGACA	This study
pMDR_4KB_R	TCTCGTCGAGGCTGTGAATG	This study

### Sanger Sequencing and Phylogenetic Analysis of the Full Length *tetC* Gene

The full-length *tetC* gene was amplified with the Phusion high fidelity enzyme (Thermo Fisher Scientific, Waltham, MA, United States) using the tetC-PF and tetC-TR primers (Table 1) with a temperature cycle of 98°C for 2 min followed by 35 cycles of 98°C for 10 s, 59°C for 30 s, 72°C for 1 min, with a final extension of 72°C for 10 min. PCR products were sent to Eton Biosciences (Research Triangle Park, NC) for sequencing. Gene sequences for *tetA-E* were retrieved from NCBI and used to build a phylogenetic tree with CLUSTAL (Sievers et al., 2011) based on sequence similarity.

### Genome Sequencing

Samples of one representative wild-type OTC*^R^* strain from each of the four orchards (F1, M1, R1, and T1) were grown overnight in nutrient broth (NB). DNA was extracted using the Wizard Genomic DNA purification kit (Promega, Madison, WI, United States) following the manufacturer’s instructions and sent for whole genome sequencing on the Illumina NextSeq 2000 system at the Microbial Genome Sequencing Center (MiGS, Pittsburg, PA, United States). DNA libraries were prepared with the Illumina DNA Prep, Tagmentation kit, with read lengths of 2 × 150 bp (Illumina Inc., San Diego, CA, United States), and bcl2fastq (v2.20.0.422)^1^ was used to demultiplex samples and trim the adapters. The raw reads were assembled with Unicycler v0.4.8 (Wick et al., 2017) utilizing its bold prediction strategy to achieve the highest contiguity.

From the Illumina sequencing, a multi-drug resistant (MDR) plasmid was found in each of the four OTC*^R^* strains, however, the plasmid in strain R1 was assembled ‘‘linear’’ instead of circular. To examine the structural variation of this plasmid in R1, we also sent R1 and T1 (as reference) for sequencing with Oxford Nanopore platform at MIGS with DNA extracted using Qiagen Blood and Tissue kit (Qiagen, Germantown, MD, United States). DNA libraries were prepared with the Genomic DNA by Ligation kit (Oxford Nanopore Technologies, New York, NY, United States) and the samples were loaded on R9 flow cells. Guppy (v 4.2.2)^2^ was used for high accuracy base calling.

The genomes of R1 and T1 were assembled to completion using the Nanopore long reads. Raw Nanopore reads for each genome were assembled with Flye v2.9 (Kolmogorov et al., 2019) utilizing customized options for a genome size of 5.2 M, one iteration of polishing, the plasmid rescue flag, and the meta flag for assembly of data with uneven coverage (Kolmogorov et al., 2020). Draft genomes were circularized using Circlator v1.5.5 (Hunt et al., 2015) with a merge minimum percent identity of 85 and a merge break length of 1000, and polished with Illumina short reads using Pilon v1.24 (Walker et al., 2014). Completeness and quality of the T1 and R1 genomes were assessed with BUSCO v5.2.2 (Simão et al., 2015) and Merqury v1.3 (Rhie et al., 2020). The k-mer databases in Merqury were built from the Illumina short reads of each strain with a k-mer size of 16.

Contig level assemblies were annotated with PROKKA v1.14.5 (Seemann, 2014), and gene clustering was performed with Panaroo v1.2.7 (Tonkin-Hill et al., 2020) using a percent identity threshold for gene clusters of 98% and Clustal (Sievers et al., 2011) to align the core genomes. Locations of the OTC and STR resistance genes were determined with a forward BLASTn of the *tetC* and *strAB* homologs from NCBI. Hypothetical proteins on the MDR plasmids were further annotated by BLASTp against the NCBI or UniProt databases. Potential structural variation among plasmids and transposons was first assessed through alignments with Clustal Omega (Sievers et al., 2011) and MAUVE (Darling et al., 2004), and later confirmed by read mapping using Hisat2 v2.2.1 (Kim et al., 2019). All formatting, sorting, and indexing of alignment files was conducted with Samtools v1.14 (Li et al., 2009).

### INDEL PCR

Raw reads from each genome were aligned to each *de novo* assembled plasmid as stated above with Bowtie2 and variations were called with Freebayes. Read alignments were visualized in Geneious (Kearse et al., 2012) and universal primers were designed with Primer3 (Untergasser et al., 2012) to amplify the indel in strains T1, M1, F1, and R1. The structural variation and the proximity of *tetR* to *tetC* in strain R1 was assessed with two pairs of primers: tetR-F/strAT-R and tetR-All-F/tetR-R (Table 1). PCR reactions were performed with strains T1 and R1 using the Phusion DNA polymerase with a temperature program of 98°C for 2 min followed by 35 cycles of 98°C for 10 s, 65°C for 15 s, 72°C for 30 s, with a final extension of 72°C for 10 min. The primers 3708F/5276R (Table 1) flanking the insertion in T1 were used with the Phusion polymerase with the same PCR program described above. PCR products of 3708F/5276R were visualized on a 1% agarose gel and then sent to Eton Biosciences for Sanger sequencing.

### Conjugation Between *Xap* and *X. perforans*

Each of the four sequenced OTC*^R^ Xap* strains were mated with *X. perforans strain* GEV1001 (Abrahamian et al., 2018) carrying a chromosomal mutation for rifampicin resistance. *Xap* and *X. perforans* were suspended in 1x PBS to an OD_600_ = 0.1, mixed at a ratio of 2:1 and grown on NA overnight at 28°C. The mixed culture was resuspended in 1x PBS (OD_600_ = 0.1) and plated on NA with 100 μg/mL of rifampicin and 25 μg/mL of OTC. The cell density (CFU/mL) of the recipient was calculated through plating dilutions of the combined culture on NA with 100 μg/mL rifampicin; the conjugation assay for each donor strain was independently performed twice. The species of transconjugants were verified with qPCR using the primers and probe (Table 1) specific for *X. perforans* (Strayer et al., 2016) or *Xap* (Palacio-Bielsa et al., 2011) using the SsoAdvanced Universal Probes Supermix at 98°C for 3 min, followed by 40 cycles of 98°C for 15 s and 69°C (for *X. perforans*) or 60°C (for *Xap*) for 60 s. Non-template control was included in each run. PCR amplification of *tetC* (primers: tetCP-F/tetCT-R), *tetR* (tetR-F/tetR-R), *strA* (strAP-F/strAT-R), *strB* (strBP-F/strBT-R) and another 4 kb fragment covering the *mobA, mazEF*, and *repA* genes (primers: pMDR_4KB_F/pMDR_4KB_R, Table 1) in the MDR plasmids were conducted with DNA from the transconjugants, *Xap* donors, and GEV1001. PCR reactions with the primers pMDR_4KB_F/pMDR_4KB_R were conducted with Phusion high fidelity enzyme with a temperature cycle of 98°C for 30 s, 35 cycles of 98°C for 10 s, 65°C for 20 s, 72°C for 3 min, and a final extension of 72°C for 10 min. PCR reactions with the other primers were as described above. Additionally, potential transfer of the ubiquitous plasmid pXap41 (Pothier et al., 2011b) from *Xap* to *X. perforans* was assessed with the pXap-41-F/pXap-41-R primers (Table 1) using the SsoAdvanced universal SYBR Green Supermix at 98°C for 3 min, and 39 cycles of 98°C for 15 s and 64°C for 30 s, and a melting curve analysis as described above.

### Cloning and Transfer of *tet* Genes Into Oxytetracycline-Sensitive *Xanthomonas* Strains

The *tetC* gene in the T1 strain was amplified using the tetCP-F and tetCT-R primers. The combined *tetC*, *tetR*, and their *cis-*regulatory elements (*tetCR*) in T1 were amplified using the tetR-xbal and tetR-kpn1 primers (Table 1). The high-fidelity PCR reactions used Phusion polymerase with a temperature program of 98°C for 2 min, 98°C for 10 s, 68°C for 10 s, and 72°C for 35 s. The PCR product was cloned into pGEM-T Easy (Promega, Madison, WI, United States) after adding poly-A tails using 2x APEX master mix (Genesee Scientific, San Diego, CA, United States). Plasmids were extracted with a ZymoPURE Plasmid Miniprep Kit (Zymo Research, Irvine, CA, United States) and the *tetC* and *tetCR* inserts were subcloned into pBBR1-MCS-2 (Bonomi et al., 2016) and transformed into Stellar competent *E. coli* cells (Takara Bio USA, Mountain View, CA, United States). The donor Stellar *E. coli* lines with the pBBR1-MCS2-tetC and pBBR1-MCS-2-tetCR plasmids were mated to the OTC- and STR-sensitive recipients *X. perforans* GEV1001 and *Xap* strain 2WF9 (a wild-type strain that was induced for rifampicin resistance on NA amended with 200 μg/mL rifampicin) (Table 1) with a chromosomal marker for rifampicin resistance. The matings were facilitated by a helper strain pRK2013 (Figurski and Helinski, 1979). All three strains were grown separately overnight in LB or NA with their corresponding antibiotics. Cells were spun down and resuspended in 1x PBS, mixed at a ratio of 2 (recipient) to 1 to 1, spot mixed on NA for overnight growth at 28°C before streaking on NA with 100 μg/mL of rifampicin and 25 μg/mL of OTC. All strains used in this experiment were identified to the species level using the qPCR assays specific for *X. perforans* (Strayer et al., 2016) or *Xap* (Palacio-Bielsa et al., 2011) as described above.

### Gene Expression Analysis of *tetC* and *tetR*

Strains T1, M1, F1, and R1 were grown for 24 h in NB and NB amended with 25 μg/mL OTC; OD_600_ values of each strain were standardized to 0.1 with NB before RNA extraction. RNA was extracted with a Zymo Quick RNA kit (Zymo Research, Irvine, CA, United States), and reverse transcription was conducted with the iScript gDNA Clear cDNA Synthesis kit (Bio-Rad Laboratories, Hercules, CA, United States). The cDNA samples were amplified for *tetC*, *tetR*, and *gyrA* (reference gene) with primers tetC-F/tetC-R, tetR-F/tetR-R, and gyrA-F/gyrA-R, respectively (Table 1). qPCR was conducted with the SsoAdvanced universal SYBR Green Supermix with 98°C for 3 min, and 39 cycles of 98°C for 15 s, 61°C for 20 s, and 72°C for 45 s, followed by a melt curve analysis described above. Standard curves for the PCR efficiency (E) of each primer set were calculated as (1 + E) = 10^(–1/slope)^ (Pfaffl, 2001) and transcript abundance was calculated with the equation (1 + E)^(–Δ^ *^Ct)^*, where ΔCt equals the Ct of the reference gene subtracted from the Ct of the target gene. The change in expression of *tetC* and *tetR* in response to OTC treatment was represented by the transcript fold differences in OTC-amended (O) samples relative to controls (C), which was calculated from the equation (1 + E_*gene*_)^(C_*Tc*_ – C_*To*_)/(1 + E_*ref*_)^(C_*Tc*_ – C_*To*_). The experiment was conducted three times. The fold changes of *tetC* and *tetR* expression in response to OTC treatment were analyzed with a generalized linear mixed model in MiniTab v. 18, with strain as the fixed effect and replication as the random effect.

## Results

### Oxytetracycline- and Streptomycin-Resistant *Xap* Strains Are Present in South Carolina Peach Orchards

**FIGURE 1:**
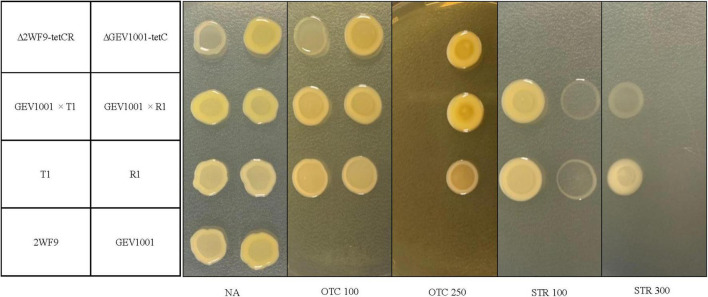
Growth of bacterial strains at 4 days after incubation on nutrient agar (NA), NA amended with 100 or 250 μg/mL of oxytetracycline (OTC 100 and OTC 250) and NA amended with 100 or 300 μg/mL of streptomycin (STR 100 and STR 300). Bacterial strains include: 2WF9 [an OTC- and STR-sensitive, rifampicin-resistant strain, *Xanthomonas arboricola* pv. *pruni* (*Xap*)], GEV1001 (an OTC- and STR-sensitive, rifampicin-resistant strain, *Xanthomonas perforans*), T1 and R1 (OTC- and STR-resistant wild-type strains, *Xap*), transconjugants with GEV1001 as recipient and T1 or R1 as donors (GEV1001 × pT1 and GEV1001 × pR1), the mutant of 2WF9 with insertion of *tetCR* genes cloned from T1 (Δ2WF9-*tetCR*), and the mutant of GEV1001 with the insertion of *tetC* gene cloned from T1 (ΔGEV1001-*tetC*). The strains were identified to the species levels using the *Xap*- and *X. perforans*-specific qPCR assays (Palacio-Bielsa et al., 2011; Strayer et al., 2016).

During 2017–2020, 430 *Xap* isolates were collected from symptomatic leaf, fruit, or twig tissues in the commercial peach orchards in 10 farms, located in six counties in South Carolina (Supplementary Table 1). Seven OTC*^R^*STR*^R^* strains (11.6% of the 2017 collection and 2.3% of the 2020 collection) with two resistant phenotypes were identified from symptomatic leaves in four peach orchards 20–270 km apart (Table 2). These OTC*^R^*STR*^R^* strains caused similar bacterial spot symptoms on inoculated peach leaves in the greenhouse compared to the sensitive *Xap* strains (Supplementary Figure 1). They account for 22.2, 1.8, 3.2, and 75% of *Xap* strains collected from Farms 1–4, respectively (Table 2 and Supplementary Table 1). Six of the seven strains exhibited the first resistant phenotype of growth on media containing OTC at concentrations up to 100 μg/mL and STR up to 300 μg/mL, while the other strain, R1, exhibited the second resistant phenotype of growth on media containing ≤250 μg/mL OTC and ≤100 μg/mL STR (Figure 1). The correlation between OTC*^R^* and STR*^R^* in *Xap* strains suggested that the genes controlling these traits are linked. All resistant strains were isolated from orchards that had been regularly treated with OTC but had never been exposed to STR.

**TABLE 2:** Oxytetracycline- (OTC) and streptomycin (STR)-resistant *Xanthomonas arboricola* pv. *pruni* (*Xap*) strains used in this study.

South Carolina County	Farm	Isolation year	Total no. of *Xap* isolates	No. of OTC^R^STR^R^ Strains^a^	OTC resistance threshold (ppm)	STR resistance threshold (ppm)
Chesterfield	1	2017	9	2	100	300
Edgefield	2	2020	55	1	250	100
Saluda	3	2020	31	1	100	300
Spartanburg	4	2017	4	3	100	300

*^a^Strains per farm were as follows, Farm 1: M1, M2; Farm 2: R1; Farm 3: F1; Farm 4: T1, T2, T3.*

### Oxytetracycline- and Streptomycin-Resistant *Xap* Strains Carry *tetC, tetR*, and the *strAB* Operon

PCR with primers that amplify known OTC and STR resistance genes were used to determine if these genes are present in the resistant strains (Table 1). We detected the *tetC, tetR*, and *strA* and *strB* genes in the OTC*^R^* and STR*^R^* strains, but not in the sensitive strains (Supplementary Figures 2, 3). Sanger sequencing of the *tetC* gene in all OTC*^R^* strains revealed 100% sequence identity among them.

**FIGURE 2:**
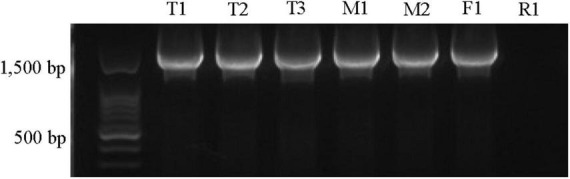
Amplification of *tetC* and *tetR* (1.9 kb amplicon) in oxytetracycline-resistant *Xanthomonas arboricola* pv. *pruni* strains. The primers tetR-Xbal/tetR-kpn1 were used. The strain IDs were labeled on top of the gel picture. The 100 bp ladder (Biotium, Fremont, CA, United States) was used. All of these strains tested positive for *Xap* using the probe-based qPCR assay (Palacio-Bielsa et al., 2011).

Phylogenetic analysis confirmed close relationship of the *Xap tetC* with previously identified *tetC* sequences in the NCBI database (Supplementary Figure 4) and revealed 99.7–100% similarities to *tetC* sequences from *Chitinibacter* sp. 2T18, *Candidatus saccharibacteria, Pseudomonas aeruginosa* and many other species. Furthermore, the orientation and proximity of *tetC* to *tetR* in all strains except R1, similar to the configuration in other bacteria, was confirmed by PCR co-amplification of *tetC* and *tetR* using one set of primers (Figure 2; Roberts, 1996; Chopra and Roberts, 2001). These results indicated that there was genetic variation in the *tetC* or *tetR* region in the R1 strain.

### Sequence Analysis Indicates That the Oxytetracycline and Streptomycin Resistance Is Plasmid-Based

**FIGURE 3:**
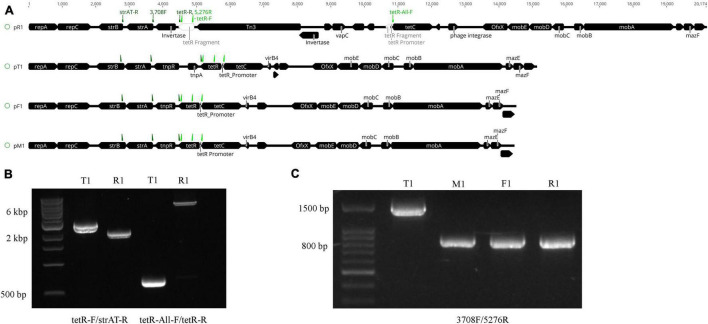
Structural variations of the plasmids harboring the oxytetracycline (OTC) and streptomycin (STR) resistance genes in *Xanthomonas arboricola* pv. *pruni*. **(A)** Linear plasmid map of the *de novo* assembled plasmids harboring the tetracycline efflux gene *tetC*, the OTC transcriptional repressor *tetR*, and the streptomycin resistance operon *strAB*. The black boxes indicate genes with the points denoting their directions. *tetR* in strain R1 was physically separated into two parts, bases 1–136 and 137–639, via the Tn*3* insertion. On each plasmid, primers strAT-R, 3708F, and tetR-R were labeled as dark green arrows from left to right, and primers tetR-F, 5276R, and tetR-All-F were labeled as light green arrows from left to right. **(B)** PCR products of DNA from strains T1 and R1 amplified with the primers tetR-F/strAT-R and tetR-All-F/tetR-R. The amplicon sizes are 2,809, 2,109, 699, and 6,969 bp from left to right. The 1 kb Extend DNA ladder (New England Biolabs) was used in the gel. **(C)** PCR products of T1, M1, F1, and R1 with the 3708F/5276R primers. The amplicon size is 1,568 bp in T1 and 869 bp in the other three strains. The 100 bp ladder (Biotium) was used in the gel. All of the strains tested positive for *Xap* using the probe-based qPCR assay (Palacio-Bielsa et al., 2011).

To gain further insight into the genetics of OTC and STR resistance, Illumina short-read sequencing was conducted for one representative OTC*^R^* strain from each peach orchard (Table 2). With these short sequencing reads, a MDR plasmid containing *tetCR* and *strAB* genes was identified in each of the four strains. This plasmid was circular in all strains except for R1 where the assembly of the plasmid was incomplete. Thus, we further sequenced strains R1 and T1 (as reference) with Nanopore technology. The Nanopore long reads helped complete the gapless genome assembly for R1 and T1. Each gapless assembly includes one circular chromosome (5,107,097 bp in R1 and 5,117,486 bp in T1), one universal circular plasmid pXap41 (41,093 bp in R1 and 41,084 bp in T1, Pothier et al., 2011b), and one circular MDR plasmid (20,174 bp in R1 and 15,112 bp in T1). Complete BUSCO scores of 99.5 and 99.6% were called for R1 and T1, respectively. The Merqury analysis generated a genome quality value of 43.9 and 42.0 with 98.1 and 99.1% of completeness for R1 and T1, respectively. The raw reads and genome assembly were deposited in Genbank under BioProject PRJNA762222, PRJNA762223, PRJNA762221, and PRJNA762226 for strain F1, M1, R1, and T1, respectively. The assembly data statistics for all four strains are shown in Table 3. Among the four strains, we observed a pan-genome of 4,606 genes and a shared core-genome of 4,146 genes, with each strain carrying the ubiquitous plasmid pXap41 (Pothier et al., 2011b). The complete assemblies of the circular MDR plasmids in these four strains were named pF1 (14,503 bp), pM1 (14,449 bp), pT1 (15,112 bp), and pR1 (20,174 bp) (Figure 3A and Supplementary Figure 5).

**TABLE 3:** Genome statistics of four oxytetracycline- and streptomycin-resistant *Xanthomonas arboricola* pv. *pruni* (*Xap*) strains.

Strain	No. of reads	No. of bases (Mbp)	Genome assembly size (bp)	No. of contigs	N50 (kbp)	GC%	CDS^a^	MDR plasmid (bp)
M1^b^	4,288,094	587.7	5,085,336	174	50	65.5	4,246	14,449
R1	76,975^c^; 2,030,284^d^	703.5^c^; 281.2^d^	5,168,364	3^e^	5,107	65.3	4,560	20,174
F1	1,707,578	237.5	5,257,079	190	43	65.3	4,434	14,503
T1	121,833^c^; 2,839,844^d^	958.7^c^; 277.3^d^	5,173,682	3^e^	5,117	65.3	4,503	15,112

*The statistics for strain F1 and M1 was based on the Illumina short read sequences only and the statistics for strain R1 and T1 was based on the combination of Nanopore long reads and Illumina short reads.*

*^a^CDS called by PROKKA.*

*^b^M1 was the only strain that was sequenced twice on Illumina NextSeq 2000 and its genome statistics was based on the combined data from both sequencing runs.*

*^c^Data from the Nanopore sequencing.*

*^d^Data from the Illumina sequencing.*

*^e^The assembly contains one complete chromosome and two circular plasmids. Complete BUSCO scores were 99.5 and 99.6% for R1 and T1, respectively. The Merqury analysis generated genome quality values of 43.9 and 42.0 with 98.1 and 99.1% of completeness for R1 and T1, respectively.*

Nanopore sequencing identified the Tn*3* transposable element inserted into the *tetR* gene in pR1. The Tn*3* element was found in the genome of the other three strains but was not present in their MDR plasmids. The ∼6 kb insertion including the Tn*3* transposase and four other genes annotated as hypothetical proteins in pR1 was confirmed by PCR with primers flanking the region (Figures 3A,B). The Tn*3* insertion separated the 5′ end of *tetR* (1–136 bp) and its promoter sequence (AGCTTTAAT, identified by BPROM) (Solovyev and Salamov, 2011) from the rest of the gene (137–503 bp) (Figure 3A and Supplementary Figure 5), which could impact *tetR* expression and the OTC resistance phenotype in strain R1. No variation was observed in the *tetR* promoter sequences between the four OTC*^R^* strains, but the C-terminus of *tetR* had sequence variation among all four strains (Supplementary Figure 6).

Aside from the Tn*3* insertion on pR1, the other major site of sequence variation between the MDR plasmids was observed in the region between *strA* and *tetC* (Figure 3A). In pT1, this region has >68% nucleotide identity with the class II transposable element Tn*5393*, which contains the *tnpA* and *tnpR* genes (Figure 3A). In the other three plasmids, *tnpA* is missing and *tnpR* is truncated. The *tnpR* gene is 100% identical among strains R1, M1, and F1, but only shared 77.5% nucleotide identity with the *tnpR* gene from strain T1. PCR with the primer 5276R that flanks *strA* and 3708F that resides in the conserved region of *tetR* (Table 1) yielded a 1,568 bp amplicon for T1 and an 869 bp amplicon for the other three strains (Figure 3C), confirming the sequence variation identified from the genome sequencing. Sanger sequencing results of these amplicons matched 100% with the sequences in the genome assembly. Unlike pR1 and pT1, the pM1 and pF1 sequences lack structural variation and share a pairwise nucleotide identity of 99.6%.

Furthermore, BLASTn searches of pT1, pM1, and pF1 in the NCBI database revealed >11,000 identical nucleotides to plasmids from *Edwardsiella tarda* strain *150611-1*, *Citrobacter freundii* strain *RHBSTW-00697*, *Klebsiella sp*. *WP7-S18-ESBL-04*, and *Aeromonas salmonicida* subsp. *salmonicida* strain *SHY16-3432*. The alignment of pT1 to *A. salmonicida* (Accession: NZ_CP038105.1) and *E. tarda* (Accession: NZ_MF925338.1) is shown in Supplementary Figure 7. Plasmid pR1 shared >9,000 bp with the above-mentioned bacteria. The main differences in gene content among these plasmids is the sequence variation in the tetracycline efflux genes and the presence of the *strAB* operon and *tnpA* and *tnpR* genes in *Xap*. The region containing the *strAB* operon in plasmid pT1 is annotated as a transposase in the *Aeromonas* and *Edwardsiella* plasmids and contributes to a significant portion of sequence variation among these species (Supplementary Figure 7).

### The Oxytetracycline and Streptomycin Resistance Genes Can Be Transferred to Oxytetracycline- and Streptomycin-Sensitive *Xanthomonas perforans* via Conjugation

To test the mobility of the OTC and STR resistance, we performed conjugation assays between the resistant *Xap* from all four geographic locations to a rifampicin-resistant (Rif*^R^*) and OTC/STR-sensitive *X. perforans* strain GEV1001 (Abrahamian et al., 2018). OTC*^R^* and STR*^R^* GEV1001 transconjugants were produced with conjugation frequencies ranging from 3.53 × 10^–7^ to 6.81 × 10^–6^ per number of recipient cells (Table 4). All GEV1001 transconjugants were able to grow on solid media with OTC and STR concentrations matching those of the donor *Xap* strains (Figure 1). The transconjugants were confirmed to be *X. perforans* via species-specific PCR (Palacio-Bielsa et al., 2011; Strayer et al., 2016). The *tetC*, *tetR*, *strAB*, and another 4 kb fragment from the MDR plasmids were successfully amplified in all the *X. perforans* transconjugants and *Xap* donors, but not in GEV1001 (Supplementary Figures 2, 3, 8). Additionally, GEV1001 transconjugants were confirmed to not harbor the ubiquitous plasmid pXap41 specifically found in *Xap* with PCR (Pothier et al., 2011b). Together, this supports that the OTC and STR resistance genes are on a mobile plasmid in *Xap* which can be transferred to other bacterial species.

**TABLE 4:** Conjugation frequencies per number of recipient cells for wild type mating assays.

Donor (oxy^R^)	Recipient (Rif^R^)	Conjugation frequency	OTC resistance in transconjugants (μ g/mL)	STR resistance in transconjugants (μ g/mL)
T1	GEV1001	1.48 × 10^–6^ to 9.39 × 10^–7^	100	300
M1	GEV1001	1.19 × 10^–6^ to 3.53 × 10^–7^	100	300
F1	GEV1001	6.81 × 10^–6^ to 6.36 × 10^–7^	100	300
R1	GEV1001	2.12 × 10^–6^ to 2.88 × 10^–6^	250	100

### Expression of *tetC* Confers Higher Level of Oxytetracycline Resistance in Sensitive Strains Than the Expression of *tetCR* Genes

To confirm gene functions, *tetC* alone and *tetCR* (the resistance gene, its transcriptional repressor, and their *cis-*regulatory elements) were separately cloned from T1 into pBBR1-MCS-2, which were then individually transferred to GEV1001 via triparental mating. The GEV1001 mutants carrying *tetCR* grew on NA amended with OTC up to 100 μg/mL, the same resistance level as seen in six of the wild-type OTC*^R^ Xap* strains, suggesting that *tetC* and *tetR* are the sole contributors to OTC resistance in these *Xap* strains (Figure 1). In contrast, when the *tetR* gene was not present, the GEV1001 mutants carrying *tetC* alone mimicked the growth patterns of strain R1 and were resistant to OTC concentrations up to 250 μg/mL (Figure 1), indicating that *tetR* is a crucial regulator and limits the level of OTC resistance in *Xap*.

### Expression of *tetC* and *tetR* Was Associated With the Oxytetracycline Resistance Phenotypes

**FIGURE 4:**
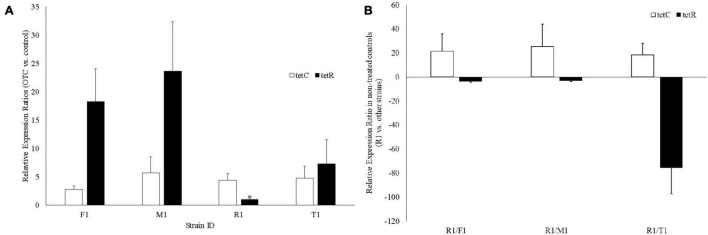
Gene expression analysis of the *tetC* and *tetR* genes in the four oxytetracycline-resistant *Xanthomonas arboricola* pv. *pruni* strains. **(A)** Fold expression changes of *tetC* and *tetR* after treatment with oxytetracycline (25 mg/L). *There was no change of *tetR* expression in R1 (relative expression ratio: 1.05, which was significantly lower than the *tetR* expression in the other three strains, *p* < 0.001); **(B)** Relative expression ratio of *tetC* and *tetR* in R1 vs. the other strains without oxytetracycline treatment.

In response to OTC treatment, *tetC* was upregulated in all four strains; *tetR* was also upregulated in all strains except in R1 where expression was not changed (Figure 4A). Without treating R1 with OTC, *tetC* was constitutively expressed (18.4–25.3-fold higher vs. the other three strains) (Figure 4B). Furthermore, R1 had significantly lower expression of *tetR* (3.1–75.5-fold) than the other three strains, which is likely responsible for the constitutive expression of *tetC* (Figure 4B).

## Discussion

This study reports and characterizes OTC resistance in a pathogenic *Xanthomonas* sp. for the first time. OTC was first registered as a pesticide in 1974 and has been used to control bacterial spot of peach since the 1980s (EPA, 1993). Despite decades of OTC application in agriculture, OTC resistance has only been reported in two plant pathogens (Spotts and Cervantes, 1995; Luo and Farrand, 1999; Hwang et al., 2005), *P. syringae* (resisting up to 500 μg/mL) and *A. tumefaciens* (resisting >20 μg/mL). In this study, *Xap* strains with two OTC*^R^* phenotypes were discovered in four orchards 20–270 km apart. In 2017, five strains with the first phenotype (22.2 and 75% of the total isolates in each orchard) were collected from two orchards. Such high percentages in a small sample size suggest that the resistant strains were prevalent or at least not rare in these two orchards at that time (Madden et al., 2017). This may have been a result of the increased selection pressure due to frequent application of OTC for bacterial spot control in peach orchards. Two different orchards were surveyed in 2020; only two resistant strains were found, one with the first phenotype and the other with the second phenotype (1.8 and 3.2% of the population within each orchard), suggesting that these strains may have recently emerged. Continued OTC application will likely maintain or even increase the prevalence of these resistant strains in commercial peach orchards (Schnabel and Jones, 1999; Chopra and Roberts, 2001; Mathers et al., 2011; Rysz et al., 2013; Kyselková et al., 2015; Abrahamian et al., 2018; Kang et al., 2018; Zhang et al., 2018). Given the limited control options available for bacterial spot, the spread of such resistant strains threatens to further increase the difficulty of bacterial spot management. This study provides a first report of the OTC and STR resistance in *Xap*. The data provided a snapshot of resistance prevalence, because each of these four orchards was only sampled once. Future studies will investigate the persistence and spread of these resistant strains in peach orchards of the southeastern United States.

The OTC resistance in our *Xap* strains was conferred by *tetC* interacting with its transcriptional repressor *tetR* on a 14–20 kb MDR plasmid that also carries *strAB* operon for STR resistance. The resistance genes on this MDR plasmid and their associated OTC*^R^*STR*^R^* phenotype were found to be transferrable to *X. perforans* via conjugation (Figure 1 and Table 4). Mobility of these plasmids into a broad range of hosts through conjugation is supported by their gene content. Replication of these plasmids may be dependent on the presence of *repA* and *repC* (Figure 3A and Supplementary Figure 5), an atypical gene cassette associated with *repABC* operon reported in *Rhizobium leguminosarum* (Cevallos et al., 2008; Pérez-Segura et al., 2013). The *repC* gene encodes a replication initiator protein, and is known to be essential for plasmid replication, whereas *repA* encodes a plasmid partitioning protein that distributes ssDNA to segregating cells (Cevallos et al., 2008; Pinto et al., 2012; Pérez-Segura et al., 2013). The formation of the relaxosome and mobilization via conjugation is likely carried out by the *mobABCDE* genes on these plasmids (Figure 3A and Supplementary Figure 5). In this cluster, *mobABC* is necessary for the initiation and termination stages of conjugal transfer while *mobD* and *mobE* act as accessory genes by drastically increasing conjugation efficiency (Perwez and Meyer, 1996; Zhang and Meyer, 1997; Bravo-Angel et al., 1999; van Zyl et al., 2003). In addition, the type IV secretion system (T4SS) located on the chromosome of xanthomonads also contributes to conjugation (Wallden et al., 2010; Guglielmini et al., 2013; Cenens et al., 2020; Alvarez-Martinez et al., 2021). Therefore, mobilization of these small plasmids is likely the outcome from the interplay of the *repAC* and *mobABCDE* genes, T4SS genes, and selection pressure from OTC sprays (Guglielmini et al., 2013). The horizontal transfer of these MDR plasmids between bacterial species poses a threat to the well-being of the peach orchards and the efficacy of current spray programs.

Lineage-specific mutations on these newly identified MDR plasmids, like the acquisition of additional antibiotic resistance genes and indels are likely the outcome of the interplay of several contributing factors, including transposons, phages, or other mobile genetic elements. Characterization of the sequence and structure variation of these plasmids is important for reporting potential differences in antibiotic resistance phenotypes. Here, we report a unique example of how a transposon insertion on the plasmid pR1 directly affected the OTC resistance levels of its host strain. The Tn*3* insertion in pR1 disrupts the *tetR* gene and helps to explain the lack of *tetR* expression, which is the most likely cause for the constitutive expression of *tetC* in strain R1, as no difference in the *tetC* promoter sequences was observed among the seven OTC*^R^* strains (Figures 3, 4). Although the *tetC* gene was upregulated upon OTC treatment in all four strains tested, the three strains without constitutive *tetC* expression may experience a shock from high OTC concentrations (>100 ppm), inhibiting their growth. In comparison, the constitutive expression of *tetC* may have protected the R1 strain from high OTC concentrations and allowed it to grow. Therefore, the observed gene expression patterns support the increased level of OTC resistance in strain R1 (same level of OTC resistance in the mutants expressing *tetCR* together) compared to the other six wild-type OTC*^R^* strains (same level of OTC resistance in the mutants expressing *tetC* alone) (Figure 1). All of these results together indicate *tetR* as the essential transcriptional regulator of *tetC* and that when *tetR* was suppressed or absent, *tetC* conferred much higher OTC resistance levels.

In addition to the structural variation in pR1, indels were also detected in the region between *strA* and *tetC* among the sequenced OTC*^R^*STR*^R^* strains (Figure 3A). The presence of transposase and resolvase genes (*tnpR* and *tnpA*) suggests the involvement of transposons in the evolution of these plasmids. This region shares >68% nucleotide identity with Tn*5393*, a class II transposable element known for harboring *tnpA* and *tnpR* and disseminating the *strAB* cassette for STR resistance, including in xanthomonads (Sundin and Bender, 1995); the main sequence variations occur in the *tnpR*, *tnpA*, and *tetCR* genes. The *tnpA* and *tnpR* genes were both found in pT1, while *tnpA* was absent and *tnpR* was truncated in the other three plasmids. A double stranded break caused by faulty transposition of the *tnpR* and *tnpA* genes that has been recorded in Tn*5393* (McManus et al., 2002) could have occurred and caused the observed deletion in pM1, pF1, and pR1. The proximity of *tetR* to this deletion site may be the cause for the sequence variation in its last nine C-terminal amino acids among our strains (Supplementary Figure 6).

The evolutionary history of these MDR plasmids is unknown, but we speculate that a potential transposon-mediated transfer of STR resistance into the tetracycline resistant plasmids could be the origin of these plasmids harboring OTC and STR resistance genes in the environmental bacteria, which was then passed on to *Xap* via conjugation. The MDR plasmids discovered in *Xap* have a similar backbone with over 73% nucleotide identity to the plasmids carrying tetracycline resistance genes in several different environmental bacteria, such as *A. salmonicida* and *E. tarda* (Supplementary Figure 7). They all carry a tetracycline efflux gene with *tetR*, and *repAC* and *mobABCDE* genes. Compared to these environmental bacteria, *Xap* strains have the additional region that contains *strAB* operon, *tnpA* and *tnpR* that resembles Tn*5393* (as discussed above). Co-inheritance of tetracycline and streptomycin resistance genes on the same plasmid has also been reported in several bacterial species in the phylloplane of apple (Schnabel and Jones, 1999). Further studies characterizing the antibiotic resistome in the peach orchards and their water supply will help identify the underlying genetic reservoirs or sources of these plasmids.

To our knowledge, this study presents the first OTC and STR resistance in *Xap* and the first OTC resistance in a *Xanthomonas* pathogen. The OTC resistance is conferred by *tetCR* genes on a small mobilizable plasmid that also harbors *strAB* operons for STR resistance, and this MDR plasmid could potentially be transferred and maintained in other bacterial epiphytes of peach in addition to *Xap*. Moreover, these plasmids are prone to structural rearrangements and mutations that could alter the OTC resistance levels, as shown in one of our strains. Further studies are needed to assess the frequency of these mutation events in field populations. Although STR is not used for disease management in peaches, the coinheritance of OTC*^R^* and STR*^R^* genotypes and phenotypes suggests that STR cannot be used as an alternative to control OTC*^R^* strains. Finally, these results emphasize a need for antibiotics resistance monitoring in United States peach orchards and development of resistance management strategies for bacterial spot.

## Data Availability Statement

The datasets presented in this study can be found in online repositories. The names of the repository/repositories and accession number(s) can be found in the article/Supplementary Material.

## Author Contributions

AH, CH, GS, JJ, XG, and HW contributed to conception and design of the experiments. AH, BC, DM, and HW collected the *Xap* isolates and conducted chemical screening. AH conducted PCR, conjugation assay, gene expression experiments, and sequence data analysis. AH and CH conducted cloning and gene transfer. RC and HW prepared samples for genome sequencing. AH and HW conducted data analysis and wrote the manuscript. AH, CH, GS, RC, JJ, MP, XG, and HW contributed to manuscript revision. HW planned the study, secured the funding, and submitted the manuscript. All authors read the manuscript and approved the submitted version.

## Conflict of Interest

The authors declare that the research was conducted in the absence of any commercial or financial relationships that could be construed as a potential conflict of interest.

## Publisher’s Note

All claims expressed in this article are solely those of the authors and do not necessarily represent those of their affiliated organizations, or those of the publisher, the editors and the reviewers. Any product that may be evaluated in this article, or claim that may be made by its manufacturer, is not guaranteed or endorsed by the publisher.

## Footnotes

1
https://emea.support.illumina.com/downloads/bcl2fastq-conversion-software-v2-20.html

2
https://nanoporetech.com/
