# Corrigendum: Oxytetracycline and streptomycin resistance genes in *Xanthomonas arboricola* pv. *pruni*, the causal agent of bacterial spot in peach

**DOI:** 10.3389/fmicb.2025.1580418

**Published:** 2025-03-17

**Authors:** Austin Herbert, C. Nathan Hancock, Brodie Cox, Guido Schnabel, Daniela Moreno, Renato Carvalho, Jeffrey Jones, Matthew Paret, Xueqing Geng, Hehe Wang

**Affiliations:** ^1^Edisto Research and Education Center, Clemson University, Blackville, SC, United States; ^2^Department of Biology and Geology, University of South Carolina Aiken, Aiken, SC, United States; ^3^Department of Plant and Environmental Sciences, Clemson University, Clemson, SC, United States; ^4^Department of Plant Pathology, University of Florida, Gainesville, FL, United States; ^5^North Florida Research and Education Center, University of Florida, Quincy, FL, United States; ^6^School of Agriculture and Biology, Shanghai Jiao Tong University, Shanghai, China

**Keywords:** antibiotic resistance, plant disease, bacterial pathogen, stone fruits, horizontal gene transfer, transposon, plasmid

In the published article, there was an error in Table 1, **page 4** as published.

In Table 1, the primer sequences for tetC-F and tetC-R are incorrect. They should instead be as so: tetC-F 5- CTTGAGAGCCTTCAACCCAG-3 and tetC-R should be 5- ATGGTCGTCATCTACCTGCC-3.

The corrected [Table 1] and its caption appear below.

**Table 1:** Bacterial strains, plasmids, and primers used in This study.

**Bacteria**	**Relevant characteristics**	**Source**
*Xanthomonas perforans* GEV1001	Rif^R^, Cu^R^	This study
Stellar Competent Cells	HST08	Takara Bio
*Xanthomonas arboricola* pv. *Pruni* 2WF9	Rif^R^	This study
Δ2WF9-tetCR	pBBR1-MCS-2-tetCR	This study
ΔGEV1001-tetC	pBBR1-MCS-2-tetC	This study
**Plasmids**		
pGEM-T Easy	Amp^R^, lacZ cloning site	Promega
pBBR1-MCS-2	Kan^R^, lacZ cloning site	Addgene
pRK2013	Kan^R^	Addgene
**Primers**		
tetC-F	CTTGAGAGCCTTCAACCCAG	Fan et al., 2007
tetC-R	ATGGTCGTCATCTACCTGCC	Fan et al., 2007
tetR-F	TTCGACGCCAAGGGATGAC	This study
tetR-R	CGTTCAAGACCGCCGATGA	This study
gyrA-F	AGGGTAACTTCGGTTCGGTC	This study
gyrA-R	CGGTTCCTGTTCCTTCTCGT	This study
tetCP-F	TCGCGAATTCTCATGTTTGACAGCT	This study
tetCT-R	TTGGCTCCAATTCTTGGAGTGGTGA	This study
strAP-F	TCATCAGAAAACTGAAGGAACCTC	This study
strAT-R	GAGTCCCGTCTGGCAATGAAA	This study
strBP-F	TTTCCTGCTCATTGGCACGTTT	This study
strBT-R	GAGGGCGAAATCCTACGCTA	This study
tetR-xba1	ATCTCTAGAGGAGGGGTTGCCCTCGATGT	This study
tetR-kpn1	ATCGGTACCTTGGCTCCAATTCTTGGAGTGGTGA	This study
tetR-All-F	CGGTGCCTGACTGCGTTAG	This study
5276R	GAAAATCGTCTACGAAGGCGGTC	This study
3708F	TCGATTCAATGGAGGTTCCTTCA	This study
FP1	CGTCGACGGCCTGGGCGA	Strayer et al., 2016
RP1	CCGGTGCCTGCGCCTGGA	Strayer et al., 2016
Xp-P	/56-FAM/CGGGCAAGG/ZEN/AGCCATCGCCTGT/3IABkFQ/	Strayer et al., 2016
Xap-2F	TGGCTTCCTGACTGTTTGCA	Palacio-Bielsa et al., 2011
Xap-2R	TCGTGGGTTCGCTTGATGA	Palacio-Bielsa et al., 2011
Xap-2P	/56-FAM/TCAATATCT/ZEN/GTGCGTTGCTGTTCTCACGA/3IABkFQ/	Palacio-Bielsa et al., 2011
pXap-41-F	ATGAAAAAGCTCTCTATCGCCCT	This study
pXap-41-R	TTCCCCCTTCTTGTTGAAATCGA	This study
pMDR_4KB_F	CCTTCATTCCGACACGGACA	This study
pMDR_4KB_R	TCTCGTCGAGGCTGTGAATG	This study

In the published article, there was an error in the **Acknowledgement section, page 11**. The acknowledgement statement containing the NIH grant ID for the Secretariat High Performance computing cluster was incorrectly displayed as “P20GM139767”. The correct NIH grant ID is “P20GM139769”.

This sentence previously stated:

“*We acknowledge Milan Panth for highly skillful technical assistance, Gary Vallad for providing us the GEV1001 strain and Clemson University for providing the Palmetto Cluster and the Secretariat High Performance computing cluster (supported by NIH grant P20GM139767) for our genome sequence analysis.”*

The corrected sentence appears below:

“*We acknowledge Milan Panth for highly skillful technical assistance, Gary Vallad for providing us the GEV1001 strain and Clemson University for providing the Palmetto Cluster and the Secretariat High Performance computing cluster (supported by NIH grant P20GM139769) for our genome sequence analysis.”*

The authors apologize for these errorrs and state that this does not change the scientific conclusions of the article in any way. The original article has been updated.

