# Supplementary material for: Oxytetracycline and Streptomycin Resistance Genes in Xanthomonas arboricola pv. pruni, the Causal Agent of Bacterial Spot in Peach
Source: Front Microbiol. 2022 Feb 25;13:821808. doi: 10.3389/fmicb.2022.821808 (PMC8914263; doi:10.3389/fmicb.2022.821808)
Supplement: Supplementary file 1 [file Table_1.docx]

**Table S1.** The number of *Xanthomonas arboricola* pv. *pruni* (*Xap*) isolates collected during 2017-2020 in South Carolina.

| **County** | **Farm** | **2017** | **2018** | **2019** | **2020** | **Total** |
| --- | --- | --- | --- | --- | --- | --- |
| Chesterfield | 1 | 9^a*^ | 72 | 22 | - | 103 |
| Edgefield | 2 | - | - | - | 55^*^ | 55 |
| Saluda | 3 | 7 | 57 | 47 | 31^b*^ | 142 |
| Spartanburg | 4 | 4^*^ | - | - | - | 4 |
| Edgefield | 5 | 5 | 24 | 7 | - | 36 |
| Greenville | 6 | 7 | - | - | - | 7 |
| Saluda | 7^c^ | 3 | 12 | 57 | - | 72 |
| Spartanburg | 8 | 5 | - | - | - | 5 |
| Spartanburg | 9 | 5 | - | - | - | 5 |
| York | 10 | 1 | - | - | - | 1 |
|  | Total | 46 | 165 | 133 | 86 | 430 |

^*^ Oxytetracycline-resistant *Xap* strains were found in the collection.

^a^ In farm 1, the peach orchard sampled in 2017 was different than the orchard sampled in 2018 and 2019.

^b^ In farm 3, the peach orchard sampled in 2020 was not sampled in the previous years.

^c^ Farm 7 is an organic peach farm where oxytetracycline had not been used for at least 15 years.
