# Supplementary material for: Oxytetracycline and Streptomycin Resistance Genes in Xanthomonas arboricola pv. pruni, the Causal Agent of Bacterial Spot in Peach
Source: Front Microbiol. 2022 Feb 25;13:821808. doi: 10.3389/fmicb.2022.821808 (PMC8914263; doi:10.3389/fmicb.2022.821808)
Supplement: Supplementary file 2 [file Image_1.PDF]

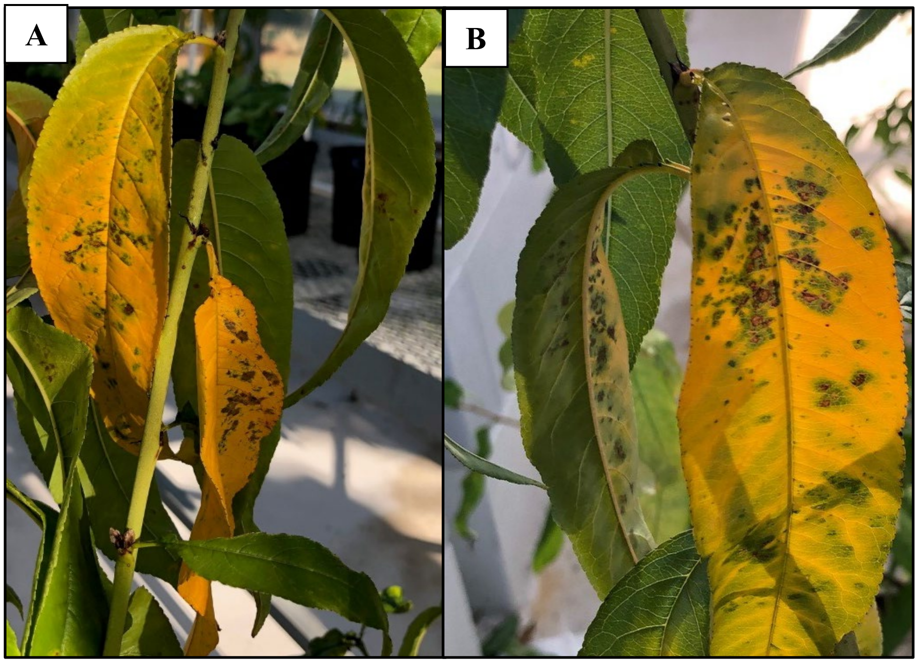

Figure S1. Bacterial spot symptoms caused by an oxytetracycline (OTC)-resistant *Xap* strain (A) and an OTC-sensitive *Xap* strain (B) four weeks after inoculation. All the OTC-resistant *Xap* strains isolated in this study caused similar bacterial spot symptoms as the sensitive *Xap* strains on inoculated peach leaves in the greenhouse.
