# Supplementary material for: Oxytetracycline and Streptomycin Resistance Genes in Xanthomonas arboricola pv. pruni, the Causal Agent of Bacterial Spot in Peach
Source: Front Microbiol. 2022 Feb 25;13:821808. doi: 10.3389/fmicb.2022.821808 (PMC8914263; doi:10.3389/fmicb.2022.821808)
Supplement: Supplementary file 4 [file Image_3.PDF]

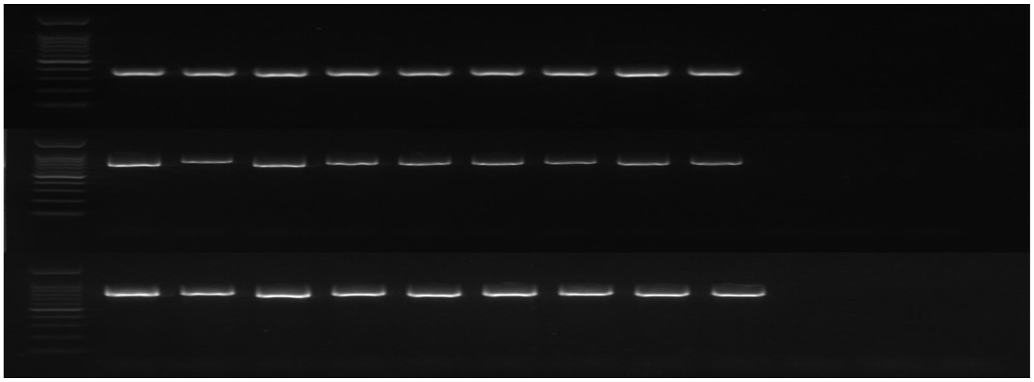

Figure S3. PCR products of the *tetR, strA* and *strB* genes with the primers tetR-F/tetR-R, strAP-F/strAT-R, and strBP-F/strBT-R in bacterial strains used in this study. Lane 1: 100 bp ladder (Biotium); lane 2-8: wild-type oxytetracycline (OTC)- and streptomycin (STR)-resistant *Xanthomonas arboricola* pv. *pruni* (Xap) strains T1, T2, T3, M1, M2, F1, R1; lane 9-10: transconjugants with *Xanthomonas perforans* strain GEV1001 as recipient and T1 and R1 as donors; lane 11-12: wildtype OTC- and STR-sensitive *Xap* strains 2WF9 and TF1(1); lane 13: OTC- and STR-sensitive GEV1001. The strains were verified to the species level using the *Xap*- and *X. perforans*-specific qPCR assays (Palacio-Bielsa et al., 2011; Strayer et al. 2016).
