# Supplementary material for: Oxytetracycline and Streptomycin Resistance Genes in Xanthomonas arboricola pv. pruni, the Causal Agent of Bacterial Spot in Peach
Source: Front Microbiol. 2022 Feb 25;13:821808. doi: 10.3389/fmicb.2022.821808 (PMC8914263; doi:10.3389/fmicb.2022.821808)
Supplement: Supplementary file 5 [file Image_4.PDF]

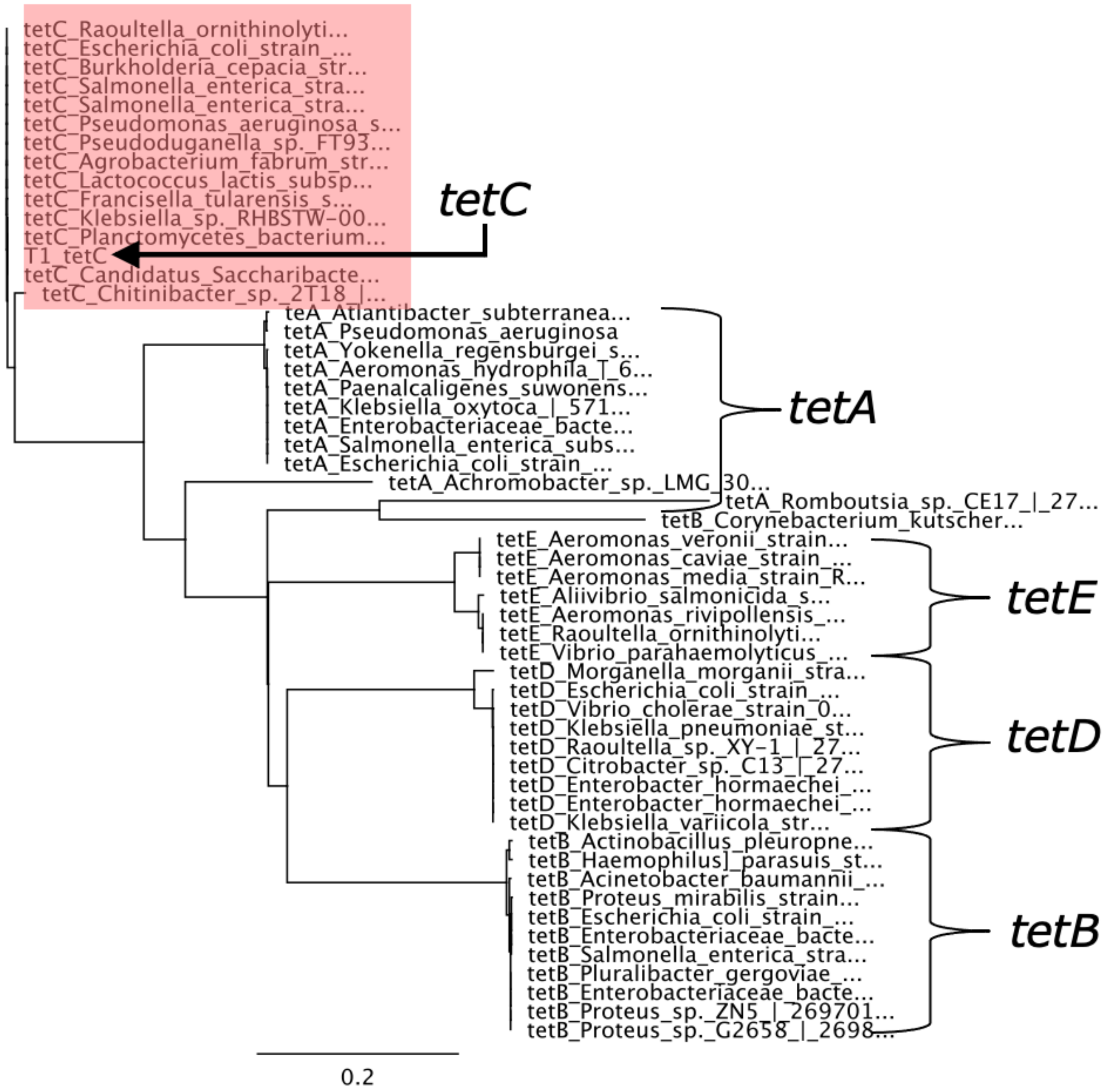

Figure S4. Phylogenetic tree of tetracycline efflux genes. Phylogenetic tree constructed from the pairwise nucleotide alignments of *tetA-E* from various Gram-positive and Gram-negative bacteria. The sequenced *tetC* gene shared 100% identity among the seven oxytetracycline-resistant strains in this study, and it is presented as T1_tetC in this graph, signaled by an arrow, and it is grouped with the other *tetC* genes.
