# Supplementary material for: Oxytetracycline and Streptomycin Resistance Genes in Xanthomonas arboricola pv. pruni, the Causal Agent of Bacterial Spot in Peach
Source: Front Microbiol. 2022 Feb 25;13:821808. doi: 10.3389/fmicb.2022.821808 (PMC8914263; doi:10.3389/fmicb.2022.821808)
Supplement: Supplementary file 6 [file Image_5.PDF]

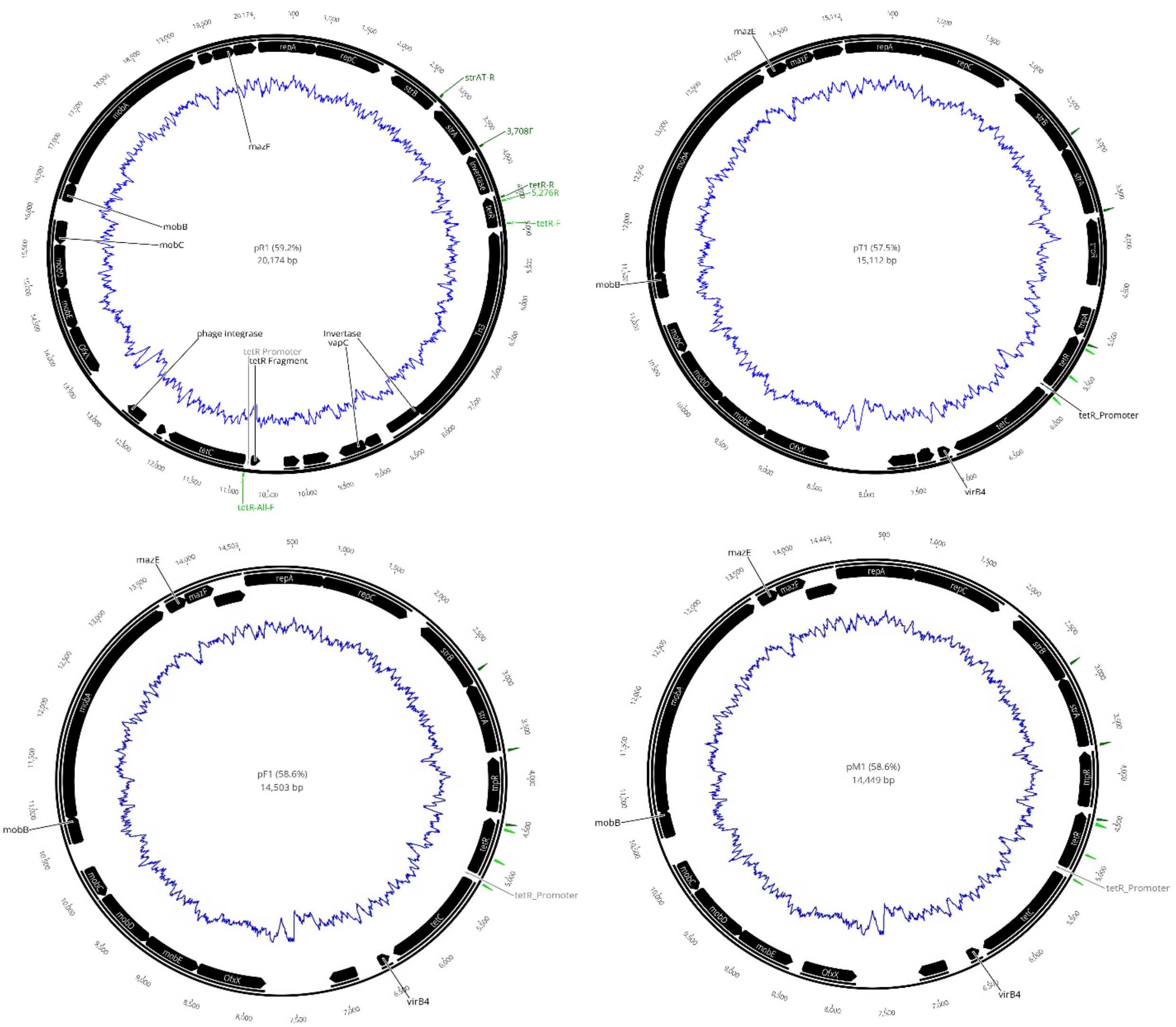

Figure S5. Circular plasmid maps of the multi-drug resistant plasmids in the four oxytetracycline (OTC)- and streptomycin (STR)-resistant strains of *Xanthomonas arboricola* pv. *pruni*. Plasmid name (pR1, pT1, pF1, or pM1) , GC% content, and size (bp) are labeled in the middle of each plasmid. The black boxes indicate genes with the points denoting their directions. Primers strAT-R, 3708F, and tetR-R (dark green arrows), tetR-F, 5276R, and tetR-All-F (light green arrows) are displayed in the clockwise order on each plasmid. The blue ring displays local GC content.
