# Supplementary material for: Oxytetracycline and Streptomycin Resistance Genes in Xanthomonas arboricola pv. pruni, the Causal Agent of Bacterial Spot in Peach
Source: Front Microbiol. 2022 Feb 25;13:821808. doi: 10.3389/fmicb.2022.821808 (PMC8914263; doi:10.3389/fmicb.2022.821808)
Supplement: Supplementary file 7 [file Image_6.PDF]

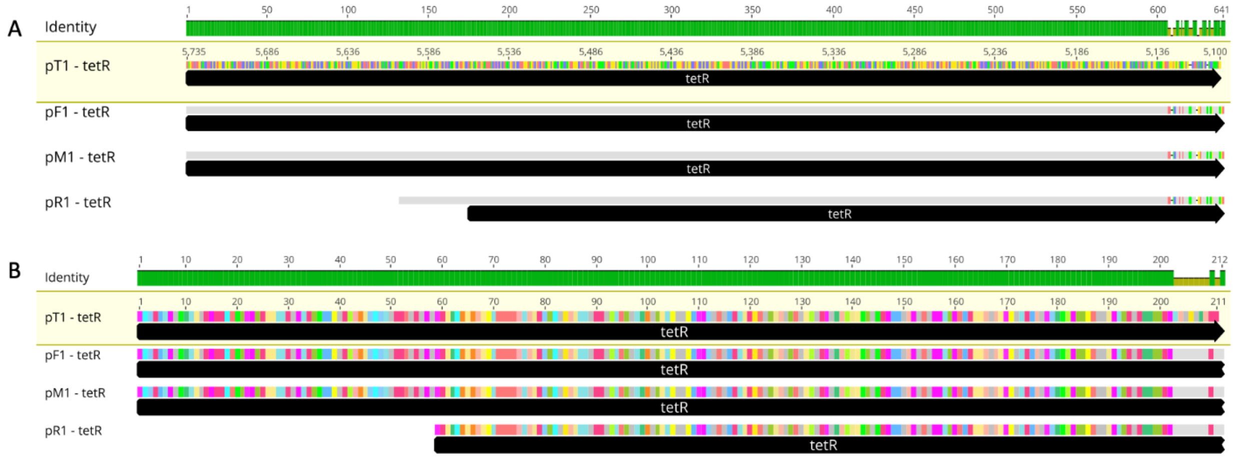

Figure S6. Nucleotide (A) and amino acid (B) alignment of *tetR* between the sequenced OTC^R strains. Protein sequences were annotated with PROKKA and are represented by the black arrow boxes in each figure.
