# Supplementary material for: Oxytetracycline and Streptomycin Resistance Genes in Xanthomonas arboricola pv. pruni, the Causal Agent of Bacterial Spot in Peach
Source: Front Microbiol. 2022 Feb 25;13:821808. doi: 10.3389/fmicb.2022.821808 (PMC8914263; doi:10.3389/fmicb.2022.821808)
Supplement: Supplementary file 8 [file Image_7.PDF]

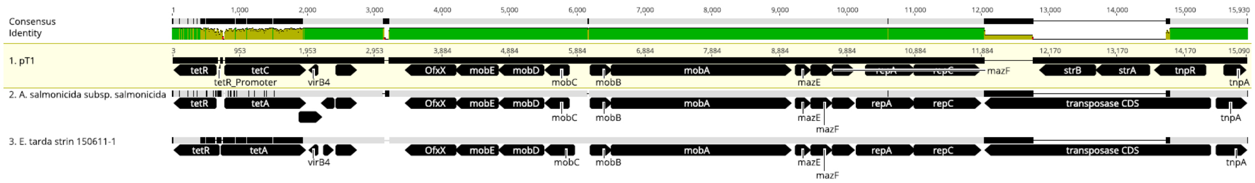

Figure S7. Pairwise nucleotide alignment of the plasmid from the oxytetracycline- and streptomycin-resistant *Xanthomonas arboricola* pv. *pruni* strain T1 to highly similar plasmids in *Aeromonas salmonicida* subsp. *salmonicida* strain *SHY16-3432* and *Edwardsiella tarda* strain *150611-1.* Genes were annotated with PROKKA. This map is not indicative of structural variation. Main differences in the gene content among species are in the tetracycline efflux genes and *strAB* operon.
