# Supplementary material for: Oxytetracycline and Streptomycin Resistance Genes in Xanthomonas arboricola pv. pruni, the Causal Agent of Bacterial Spot in Peach
Source: Front Microbiol. 2022 Feb 25;13:821808. doi: 10.3389/fmicb.2022.821808 (PMC8914263; doi:10.3389/fmicb.2022.821808)
Supplement: Supplementary file 9 [file Image_8.PDF]

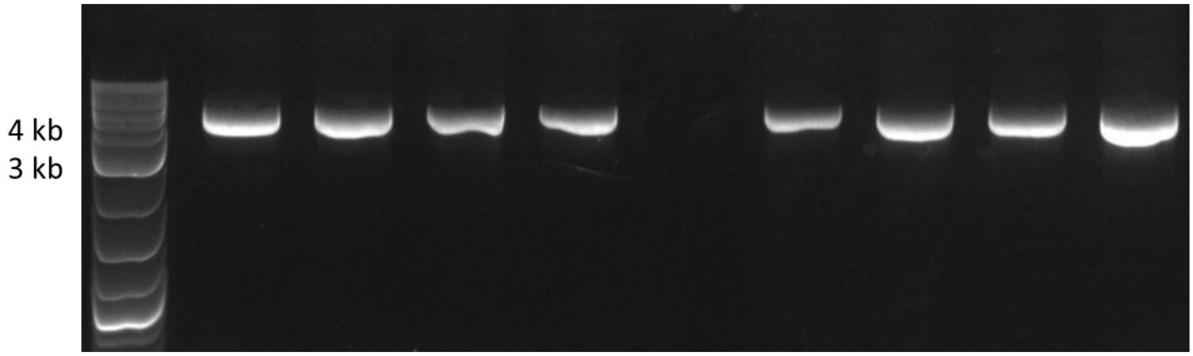

Figure S8. PCR products of a 4 kb fragment covering *mobA*, *mazEF*, and *repA* with the primers pMDR_4KB_F/pMDR_4KB_R in bacterial strains used in this study. Lane 1: 1 kb plus DNA ladder (New England Biolab); lane 2-5: wild-type oxytetracycline (OTC)- and streptomycin (STR)-resistant *Xanthomonas arboricola* pv. *pruni* (*Xap*) strains R1, T1, F1, M1; lane 6: OTC- and STR-sensitive *X. perforans* strain GEV1001; lane 7-10: transconjugants with *Xanthomonas perforans* strain GEV1001 as recipient and R1, T1, F1, or M1 as donor, respectively. The strains were verified to the species level using the *Xap*- and *X. perforans*-specific qPCR assays (Palacio-Bielsa et al., 2011; Strayer et al. 2016).
